# Clinical effect of unilateral biportal minimally invasive surgery in the treatment of patients with spinal degenerative diseases based on intelligent multimodal reconstruction technology

**DOI:** 10.3389/fmed.2025.1615699

**Published:** 2025-07-10

**Authors:** Quan Sun, Lei Wang, Jun Ma, Fei He, Dongyu Wei, Chunming Si

**Affiliations:** Department of Orthopaedic Center, Xinjiang Production and Construction Corps Hospital, Urumqi, China

**Keywords:** spinal degenerative diseases, unilateral biportal endoscopy, intelligent multimodal reconstruction technology, lumbar function, pain

## Abstract

**Objective:**

To explore the effect of unilateral biportal minimally invasive surgery in the treatment of patients with spinal degenerative diseases based on intelligent multimodal reconstruction technology.

**Methods:**

A total of 100 patients with spinal degenerative diseases treated with unilateral biportal endoscopy during 2023–2024 in Orthopedics Center of our hospital were selected as research objects. Patients using intelligent multi-modal reconstruction technology were included as observation group, and patients not using intelligent multi-modal reconstruction technology were included as control group. The length of hospital stay, operation time, intraoperative blood loss, postoperative drainage volume, total blood loss, hidden blood loss, hematocrit, hemoglobin level, incidence of complications, degree of pain and lumbar function were assessed.

**Results:**

Compared to the control group, the observation group had shorter operation time, shorter length of hospital stay, less intraoperative blood loss, less postoperative drainage volume, less total blood loss, less hidden blood loss, higher hematocrit and higher hemoglobin level (*p* < 0.01). Relative to the control group, the observation group had lower incidence of complications (*p* < 0.05). Compared with 1 day after surgery, the Visual Analog Scale score and Oswestry Disability Index score in both groups were gradually declined at 5, 10, and 15 days after surgery (*p* < 0.05). Relative to the control group, the observation group had lower Visual Analog Scale score and Oswestry Disability Index score at 5, 10, and 15 days after surgery (*p* < 0.05). Compared with 1 day after surgery, the Japanese Orthopaedic Association score in both groups was gradually elevated at 5, 10, and 15 days after surgery (*p* < 0.05). Relative to the control group, the observation group had higher Japanese Orthopaedic Association score at 5, 10, and 15 days after surgery (*p* < 0.05). Compared with 1 month after surgery, the Visual Analog Scale score and Oswestry Disability Index score were gradually decreased while the Japanese Orthopaedic Association score was gradually elevated in both groups 3, 6 and 12 months after surgery (*p* < 0.05). Relative to the control group, the observation group had lower Visual Analog Scale score and Oswestry Disability Index score as well as higher Japanese Orthopaedic Association score 1, 3, 6 and 12 months after surgery (*p* < 0.05).

**Conclusion:**

Unilateral biportal minimally invasive surgery based on intelligent multimodal reconstruction technology can accelerate the body recovery, reduce the incidence of complications, reduce the degree of pain and improve the lumbar function in the treatment of patients with spinal degenerative diseases.

## Introduction

With the progress of The Times and the rapid development of modern society, the level of social medical treatment is also accelerating the pace of development, our national average life gradually increased, the society is gradually entering the aging. As a result, the incidence and prevalence of spinal degenerative diseases are increasing year by year, for which neck and back pain are the most common clinical symptoms (1). Surgery is an important treatment for spinal degenerative diseases (2). However, traditional surgery has problems such as greater trauma, higher risk, greater surgical cost, slower postoperative recovery, and low patient recognition (3). In recent years, the development of single-channel endoscopic technology has solved many spinal diseases, but single-channel endoscopic technology has disadvantages such as expensive and vulnerable surgical instruments, long and steep technical learning curve, and intraoperative narrow field of vision, which makes it very difficult to promote the technology to the grass root level (4). However, the emergence of unilateral biportal endoscopy (UBE) has solved this problem well (5).

UBE technology refers to the establishment of two minimally invasive channels on the same side of spinal surgery, namely “observation channel and operation channel” (6). Compared with single-channel endoscopic technology, this technology has the following advantages: (1) During the operation, the field of vision is larger, and it can easily cross the midline to the contralateral side, and complete the bilateral decompression of the spinal canal (7); (2) The endoscopic channel does not interfere with the instrument channel, and the instrument is not bound by the hard pipe (8); (3) The learning curve is smooth, the surgical method is close to the open surgery minimally invasive, and it is easier for physicians to master the relevant technical points (9); (4) Without the limitation of pipe diameter, basic surgery does not require special customized tools, only a set of arthroscopic system and conventional surgical instruments can be carried out in basic hospitals (10). However, UBE technology is still difficult to learn, and local magnification of endoscopic surgery is easy to lose the overall anatomical structure. Inexperienced doctors need to coordinate the depth and direction of the endoscope for a long time, and the surgeon needs to have good three-dimensional spatial orientation (11). Therefore, if the preoperative accurate planning is required, the intraoperative positioning is efficient and safe. Providing real-time anatomical structure identification and spatial position information to assist surgery can greatly reduce the learning curve and improve surgical safety and efficiency, which is the core issue of the promotion and application of UBE technology.

In this study, we compared the therapeutic effect of unilateral biportal minimally invasive surgery on patients with spinal degenerative diseases under the conditions of preoperative guidance without intelligent multimodal reconstruction technology and under the conditions of preoperative guidance with this technology.

## Data and methods

### Patients

This was a retrospective study. In this study, a total of 100 patients with spinal degenerative diseases treated with UBE during 2023–2024 in Orthopedics Center of our hospital were selected as research objects. Patients using intelligent multi-modal reconstruction technology were included as observation group, and patients not using intelligent multi-modal reconstruction technology were included as control group. Each group had 50 patients. Inclusion criteria: (1) Patients undergoing minimally invasive spinal surgery with UBE technique due to degenerative spinal diseases; (2) Age ≥40; (3) No gender preference; (4) No limitation on nationalities. Exclusion criteria: (1) the operation area was a secondary operation; (2) abnormal bone development and bone metabolism; (3) patients with severe osteoporosis (T-value ≤ −2.5); (4) abnormal coagulation function. This study was conducted in accordance with the Declaration of Helsinki and approved by the Ethics Committee of our hospital. All participants provided written informed consent prior to enrollment.

### Randomization and blinding

A group randomization design was adopted for random grouping. The random allocation sequence was generated by a computer. The allocation confidentiality measures were achieved through sequential numbering, sealing, and opaque envelopes. After being deemed to meet the inclusion criteria, patients were randomly assigned to the control group or the observation group in a 1:1 ratio. This study was single-blind, and the participants were unaware of the allocation.

### Preoperative planning

Both the observation group and the control group underwent thin-slice computed tomography and other routine preoperative examinations before surgery, and completed the preoperative evaluation of the Japanese Orthopaedic Association (JOA) score (12), the Oswestry Disability Index (ODI) questionnaire (13), and the Visual Analog Scale (VAS) score (14). In the observation group, based on intelligent multi-mode reconstruction technology, computed tomography data were used to generate a three-dimensional reconstruction model of the spine, obtain clear anatomical information of the surgical site, complete personalized pre-operative planning and surgical rehearsal on the three-dimensional model, and obtain information such as the location of the anchor point of the operation, the scope of surgical decompression, and the best surgical process. In the control group, the scope and procedure of operation were determined by experience based on the preoperative examination according to the conventional UBE surgical method.

### Surgical method

All patients underwent UBE surgical method. The patient was put under general anesthesia and placed in prone position. After the operative area was disinfected, the “Corps double-channel water diversion method” was used to lay towels and apply film to seal the water, and the position of the responsibility gap was determined by fluoroscopy. In the observation group, according to the preoperative surgical plan, the double-channel path was established, and the anchor was found. According to the preoperative plan, laminectomy, ligamentum flavum resection, lateral recess decompression, and intervertebral disc removal were performed. In the control group, a double-channel pathway was established according to the experience of the surgeon, and the anatomical structure of the interlaminar space was found under the microscope. According to the degree of intravertebral canal nerve relaxation under the microscope, laminectomy, ligamentum flavum resection, lateral recess decompression, and intervertebral disc removal were performed. After that, according to the needs of the patient and the requirements of the patient, the intervertebral space bone graft fusion and pedicle screw fixation were performed. A negative pressure indwelling drainage tube was placed on the wound, the incision was sutured layer by layer, and the dressing was bandaged.

### Postoperative management

All patients underwent postoperative management. Routine antibiotics were used to prevent infection for 24–48 h, intravenous non-steroidal drugs were used to relieve pain for 72 h, and the drainage tube was removed within 72 h or when the drainage volume was <50 mL. Active flexion and extension of ankle joint and straight leg elevation exercises were performed after anesthesia. From 24 to 28 h after surgery, weight-bearing walking began.

### Observation indicators

(1) The length of hospital stay, operation time, intraoperative blood loss, postoperative drainage volume, hematocrit (Hct), hemoglobin (Hb), total blood loss (TBL), and hidden blood loss (HBL) were recorded in both groups. HBL = TBL − (Visible blood loss + Allogeneic blood transfusion volume). TBL = Preoperative blood volume × (Preoperative hematocrit − Postoperative hematocrit)/Average Hct.
(2) The total incidence of complications including incision infection, hematoma compression, cerebrospinal fluid leakage and lower limb numbness was recorded and compared between the two groups.
(3) The VAS score was used to evaluate the pain of patients 1, 5, 10, and 15 days after surgery. 0 points represented no pain, 1 to 3 points represented mild pain, 4 to 5 points represented moderate pain, and 6 to 10 points represented severe pain.
(4) The JOA score was used to evaluate the functional disorder of patients 1, 5, 10, and 15 days after surgery. The total score was 29 points, with the lower score indicating more obvious of functional disorder.
(5) The ODI was used to evaluate the lumbar function of patients 1, 5, 10, and 15 days after surgery, including 10 items such as standing, self-care, sitting, disturbed sleep, walking, pain intensity, sexual life, extracts, social life, and travel. The score range was 0–50 points for each item. The score was inversely proportional to lumbar function.
(6) The patients were followed up 1, 3, 6, 12 months after surgery, and JOA, ODI, and VAS scores were assessed.

### Statistical analysis

GraphPad Prism 10.0 statistical software was employed for analyzing the data. The measurement data were exhibited by mean ± standard deviation (x ± s). Normality and variance equality were tested using Shapiro–Wilk tests and Levene’s Test for Equality of Variances, respectively. Comparisons were performed with the t-test, following the assessment of normality and equality of variances. The counting data were exhibited as number and rate (%), and χ^2^ test was applied for comparison. *p* < 0.05 was considered as statistically significant.

## Results

### General data of patients in both groups

No statistical differences were seen in general data of patients between the two groups (*p* > 0.05, Table 1).

**Table 1:** General data of patients in both groups.

Groups	*N*	Gender		Age (years)	Course of disease (years)	Type of diseases			
		Male	Female			Lumbar spondylolisthesis	Degenerative lumbar instability	Lumbar disc herniation	Lumbar spinal stenosis
Control group	50	27 (54.00)	23 (46.00)	51.33 ± 13.25	5.06 ± 0.48	9 (18.00)	5 (10.00)	30 (60.00)	6 (12.00)
Observation group	50	25 (50.00)	25 (50.00)	51.36 ± 13.32	5.13 ± 0.54	8 (16.00)	3 (6.00)	32 (64.00)	7 (14.00)
χ^2^/t			0.16	0.01	0.68				0.70
*P*			0.68	0.99	0.49				0.87

### Surgery-related indexes between 2 groups

**Figure 1:**
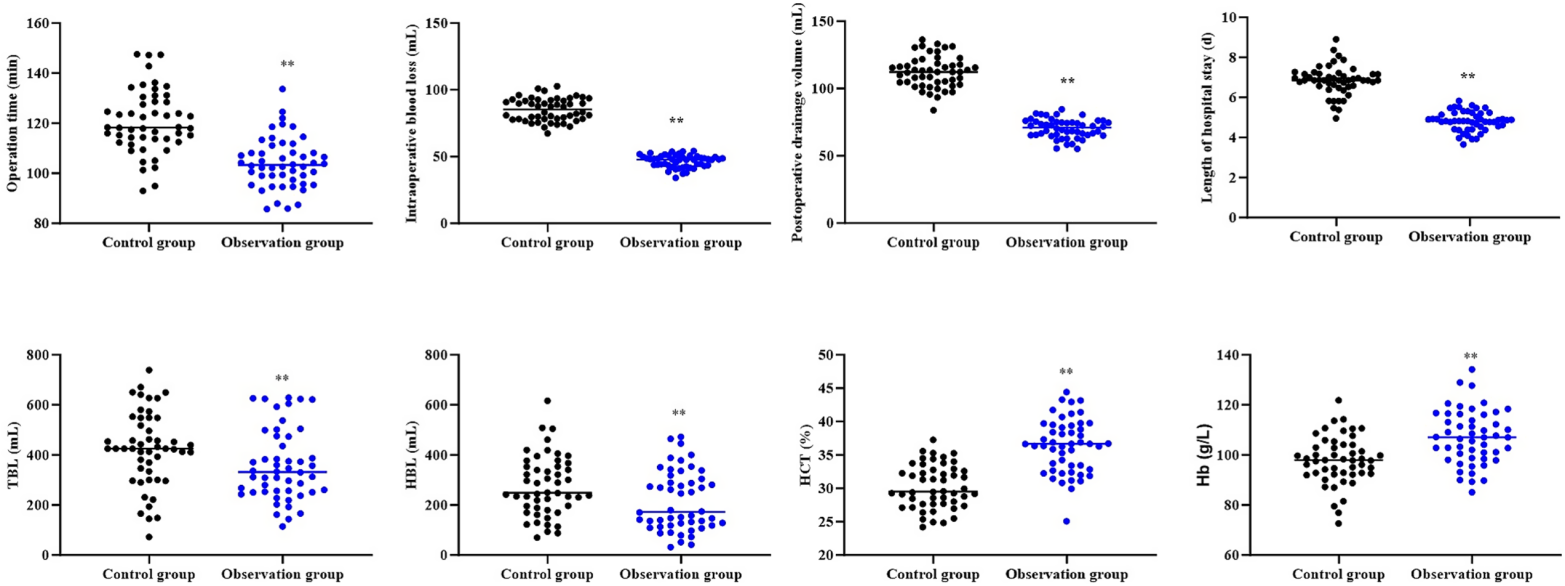
Surgery-related indexes between 2 groups. ^**^p < 0.01.

Compared to the control group, the observation group had shorter operation time, shorter length of hospital stay, less intraoperative blood loss, less postoperative drainage volume, less TBL, less HBL, higher HCT and higher Hb level (*p* < 0.01, Figure 1).

### Incidence of complications between 2 groups

Relative to the control group, the observation group had lower incidence of complications (*p* < 0.05, Table 2).

**Table 2:** Incidence of complications between 2 groups.

Groups	*N*	Incision infection	Hematoma compression	Cerebrospinal fluid leakage	Lower limb numbness	Total incidence rate
Control group	50	3 (6.00)	3 (6.00)	1 (2.00)	2 (4.00)	9 (18.00)
Observation group	50	0 (0.00)	1 (2.00)	0 (0.00)	1 (2.00)	2 (4.00)
χ^2^						5.00
*P*						0.02

### Degree of pain at different time points in both groups

**Figure 2:**
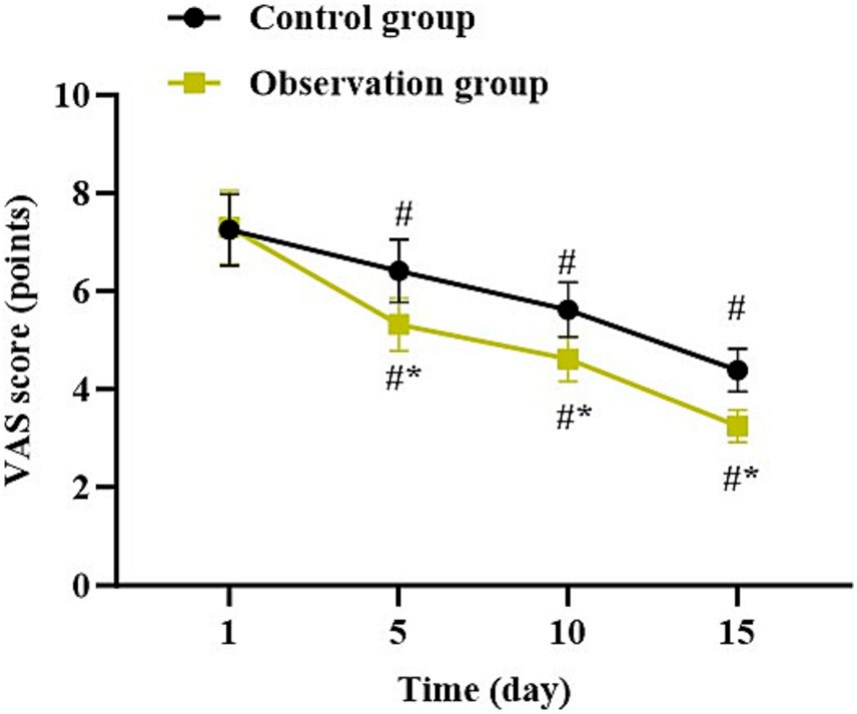
Degree of pain at different time points in both groups. ^*^p < 0.05, vs. control group; ^#^*p* < 0.05, vs. 1 day after surgery.

Compared with 1 day after surgery, the VAS score in both groups was gradually declined at 5, 10, and 15 days after surgery (*p* < 0.05). Relative to the control group, the observation group had lower VAS score at 5, 10, and 15 days after surgery (*p* < 0.05, Figure 2).

### Functional disorder at different time points in both groups

**Figure 3:**
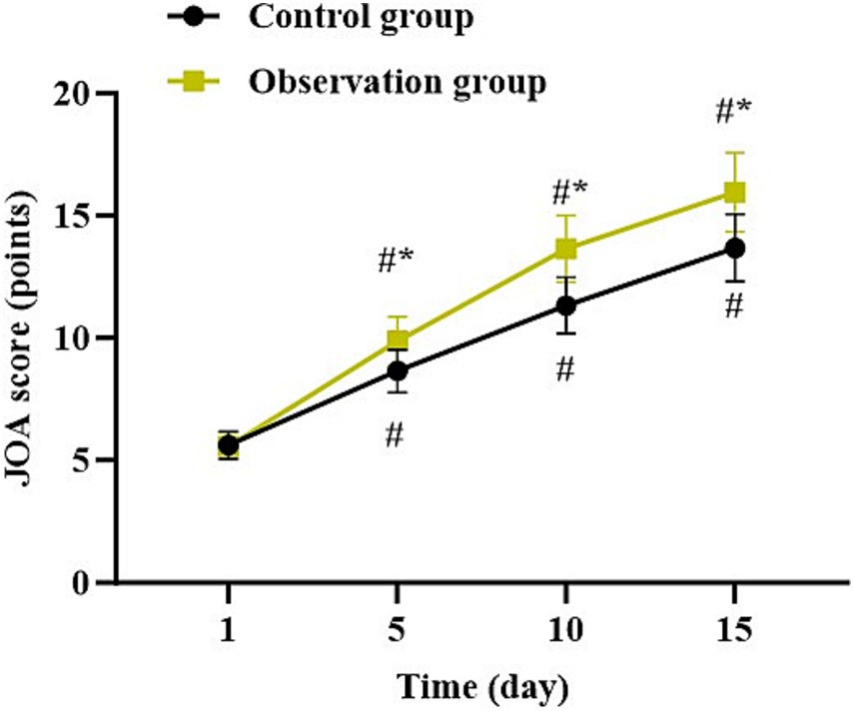
Functional disorder at different time points in both groups. ^*^p < 0.05, vs. control group; ^#^*p* < 0.05, vs. 1 day after surgery.

Compared with 1 day after surgery, the JOA score in both groups was gradually elevated at 5, 10, and 15 days after surgery (*p* < 0.05). Relative to the control group, the observation group had higher JOA score at 5, 10, and 15 days after surgery (*p* < 0.05, Figure 3).

### Lumbar function at different time points in both groups

**Figure 4:**
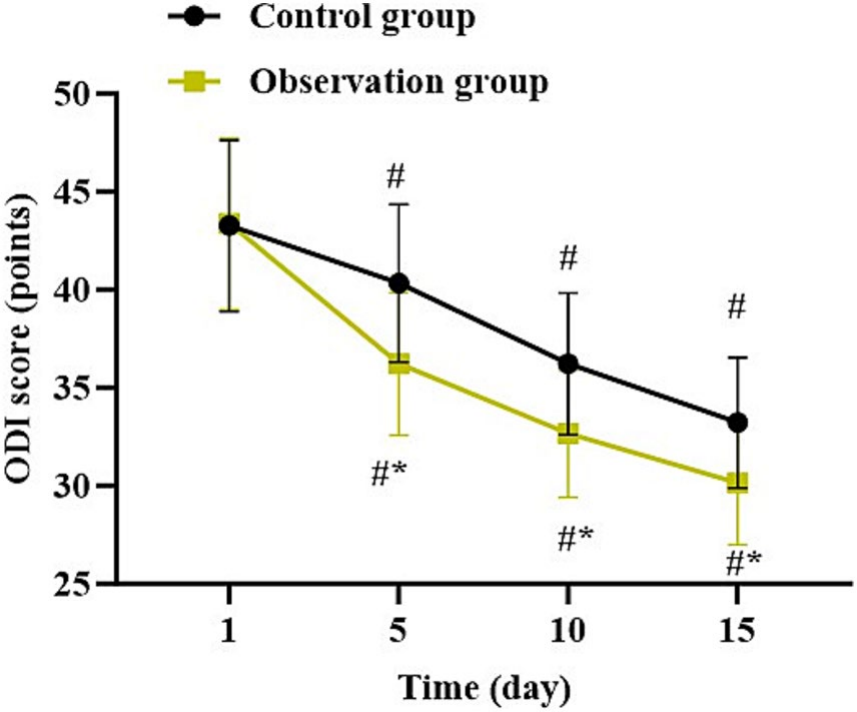
Lumbar function at different time points in both groups. ^*^p < 0.05, vs. control group; ^#^*p* < 0.05, vs. 1 day after surgery.

Compared with 1 day after surgery, the ODI score in both groups was gradually declined at 5, 10, and 15 days after surgery (*p* < 0.05). Relative to the control group, the observation group had lower ODI score at 5, 10, and 15 days after surgery (*p* < 0.05, Figure 4).

### Follow-up results of patients in both groups

**Figure 5:**
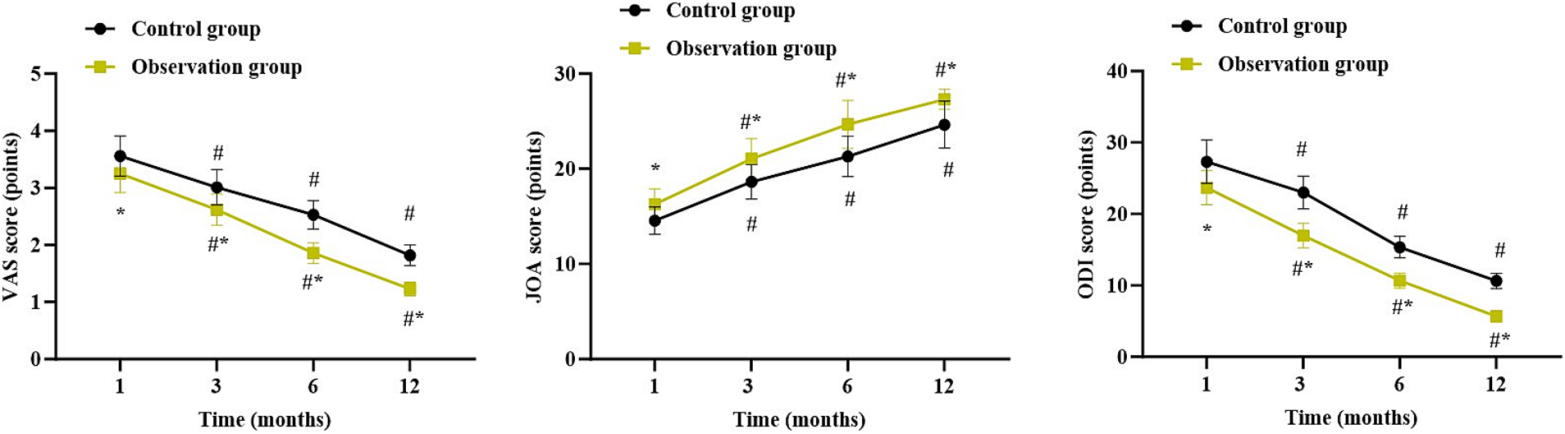
Follow-up results of patients in both groups. ^*^p < 0.05, vs. control group; ^#^*p* < 0.05, vs. 1 month after surgery.

Compared with 1 month after surgery, the VAS score and ODI score were gradually decreased while the JOA score was gradually elevated in both groups 3, 6 and 12 months after surgery (*p* < 0.05). Relative to the control group, the observation group had lower VAS score and ODI score as well as higher JOA score 1, 3, 6 and 12 months after surgery (*p* < 0.05, Figure 5).

## Discussion

The intelligent 3D reconstruction and segmentation system of complex spinal model is to study the 3D reconstruction method based on medical information and the corresponding medical application by realizing a 3D visualization environment of medical image suitable for surgical clinical diagnosis, so as to provide a platform for users to apply the system for related medical auxiliary diagnosis or practical medical surgery (15). In our study, an intelligent spinal segmentation model was applied. Firstly, the body regions were automatically identified from the images, and then the spinal-related bones were segmented and classified for recognition. Based on the rapid coarse segmentation results, the three-dimensional anatomical features of the spine and the 3D-tracing intelligent correction algorithm were utilized to obtain the final precise three-dimensional segmentation results and each vertebra segment was accurately identified. The automatic recognition algorithm is currently available in the PVmed-iCurve product and has obtained a Class III certification. By leveraging massive data, including multi-center, multi-resolution, and multi-site CT data, training with a deep learning convolutional neural network model, and extracting image features at multiple scales, a precise body region segmentation algorithm was achieved. Because the vertebral bodies cover the areas from the neck to the pelvis of the human body, and there are differences in the organs and anatomical structures at different levels of the human body, so by using the extracted body regions, the overall CT cross-sectional anatomical information was used to train the deep learning model to obtain the vertebral body regions and their labels at each layer. Because global information of the human body region needed to be utilized, a self-learning transformer model based on global information was adopted, combined with multi-scale extraction of image information to train the network model. Moreover, since continuous slices have anatomical characteristics, each layer of the continuous multi-slice input was used for prediction of the overall structure, and the gold standard was used as a discriminator to distinguish its authenticity to further improve the segmentation accuracy of the model. Although the above algorithm design can already yield relatively accurate segmentation results, in order to further improve the segmentation accuracy and ensure the accuracy of the three-dimensional anatomy, the 3D-Tracing algorithm was utilized to perform 3D structure fine-tuning along the center line of the segmented vertebrae, thereby ensuring the high robustness of the algorithm.

With the development of medical science and computer science, clinical medicine has put forward higher requirements for image processing technology. As an important image processing arm, image registration technology is widely used and of great significance in clinical practice (16). For example, it can be applied to assist disease diagnosis, monitoring of lesion morphological changes, image-guided surgical treatment and evaluation of therapeutic effects (17). To align the two-dimensional perspective images with the three-dimensional CT images, the first step was to establish the perspective process of the C-Arm, which is a classic ray-tracing algorithm (18). Through the well-known geometric calibration algorithm in the industry, the perspective model of the C-Arm was obtained. The vertebral body segmentation based on perspective images and the segmentation based on 3D CT images were achieved by collecting a large amount of data and training with the most advanced deep learning models available at present. Based on the 2D and 3D vertebral structure segmented by deep learning, through the constructed ray-tracing model and their similarity evaluation model, the initial deformation parameters were directly predicted by training the deep learning model. Through data augmentation, we could design different machine parameters and shooting angles. Through simulation, we could build a large amount of data, thereby obtaining a robust model structure. Then, using the Nelder–Mead Optimization Method (19), we could iteratively obtain the final deformation parameters to avoid the model from deviating due to some abnormal situations.

In our study, the results suggested that compared to the control group, the observation group had shorter operation time, shorter length of hospital stay, less intraoperative blood loss, less postoperative drainage volume, less TBL, less HBL, higher HCT, higher Hb level, lower VAS score at 5, 10, and 15 days after surgery, as well as lower incidence of complications. All these results suggested that UBE based on intelligent multimodal reconstruction technology could accelerate the body recovery and reduce the postoperative pain of patients with spinal degenerative diseases. Besides, our study indicated that relative to the control group, the observation group had higher JOA score and lower ODI score at 5, 10, and 15 days after surgery, suggesting that UBE based on intelligent multimodal reconstruction technology could improve the lumbar function of patients with spinal degenerative diseases. Additionally, follow-up studies showed that relative to the control group, the observation group had lower VAS score and ODI score as well as higher JOA score 1, 3, 6 and 12 months after surgery, suggesting the long-term effectiveness of UBE based on intelligent multimodal reconstruction technology. Consistent with our findings, Wu et al. suggested that the artificial intelligence-assisted surgical coaching program effectively improved surgical performance and safety for novice surgeons in laparoscopic cholecystectomy procedures (20). The reason is that through the intelligent 3D model reconstruction and segmentation system, doctors can clearly obtain the anatomical information of the patient’s surgical site and formulate personalized surgical plans (21). At the same time, the preoperative surgical planning based on the 3D model reduces the intraoperative risk, and the preoperative planning information is mapped into the endoscopic video stream to assist doctors in conducting clinical surgical operations, thereby improving the surgical efficiency, reducing the pain, and improving the lumbar function (22).

The intelligent multimodal reconstruction and the existing spinal navigation technologies have significant differences in data fusion methods, functional characteristics and application effects. Its advantages include rich information expression, high positioning accuracy, and convenient operation. However, its disadvantages include complex data acquisition and processing, difficult model training, and high hardware requirements (23). In the future, improvements need to be made in data quality, model optimization, and hardware upgrading.

Our research has some limitations. Firstly, our sample size is relatively small, which may lead to deviations between the data results and the actual values. Secondly, our research adopted a single-blind design, which inevitably resulted in subjective biases from the researchers, leading to an imbalance in the treatment between the two groups. Thirdly, our research was a single-center study, and the sample was not representative, which may not accurately reflect the characteristics of a broader population. Fourthly, our research only conducted 1-year follow-up observations, and the long-term prognosis of patients after surgery remains unclear. Therefore, more multi-center, double-blind, large-scale and long-term studies should be conducted in the future to further verify our findings.

## Conclusion

Our study demonstrates that UBE based on intelligent multimodal reconstruction technology can accelerate the body recovery, reduce the incidence of complications, reduce the degree of pain and improve the lumbar function in the treatment of patients with spinal degenerative diseases.

## Data Availability

The datasets presented in this study can be found in online repositories. The names of the repository/repositories and accession number(s) can be found in the article/Supplementary material.
